# Orexin Signaling: A Complex, Multifaceted Process

**DOI:** 10.3389/fncel.2022.812359

**Published:** 2022-04-13

**Authors:** Natasha C. Dale, Daniel Hoyer, Laura H. Jacobson, Kevin D. G. Pfleger, Elizabeth K. M. Johnstone

**Affiliations:** ^1^Molecular Endocrinology and Pharmacology, Harry Perkins Institute of Medical Research and Centre for Medical Research, The University of Western Australia, Nedlands, WA, Australia; ^2^Australian Research Council Centre for Personalised Therapeutics Technologies, Melbourne, VIC, Australia; ^3^Australian Research Council Centre for Personalised Therapeutics Technologies, Perth, WA, Australia; ^4^Florey Institute of Neuroscience and Mental Health, Parkville, VIC, Australia; ^5^Department of Biochemistry and Pharmacology, School of Biomedical Sciences, Faculty of Medicine, Dentistry and Health Sciences, The University of Melbourne, Parkville, VIC, Australia; ^6^Department of Molecular Medicine, The Scripps Research Institute, La Jolla, CA, United States; ^7^Melbourne Dementia Research Centre, Florey Institute of Neuroscience and Mental Health, The University of Melbourne, Parkville, VIC, Australia; ^8^Dimerix Limited, Nedlands, WA, Australia; ^9^School of Biomedical Sciences, The University of Western Australia, Nedlands, WA, Australia

**Keywords:** orexin, orexin 1 receptor, orexin 2 receptor, signaling, G protein, GPCR (G protein coupled receptor), arrestin

## Abstract

The orexin system comprises two G protein-coupled receptors, OX_1_ and OX_2_ receptors (OX_1_R and OX_2_R, respectively), along with two endogenous agonists cleaved from a common precursor (prepro-orexin), orexin-A (OX-A) and orexin-B (OX-B). For the receptors, a complex array of signaling behaviors has been reported. In particular, it becomes obvious that orexin receptor coupling is very diverse and can be tissue-, cell- and context-dependent. Here, the early signal transduction interactions of the orexin receptors will be discussed in depth, with particular emphasis on the direct G protein interactions of each receptor. In doing so, it is evident that ligands, additional receptor-protein interactions and cellular environment all play important roles in the G protein coupling profiles of the orexin receptors. This has potential implications for our understanding of the orexin system’s function *in vivo* in both central and peripheral environments, as well as the development of novel agonists, antagonists and possibly allosteric modulators targeting the orexin system.

## Introduction

**FIGURE 1:**
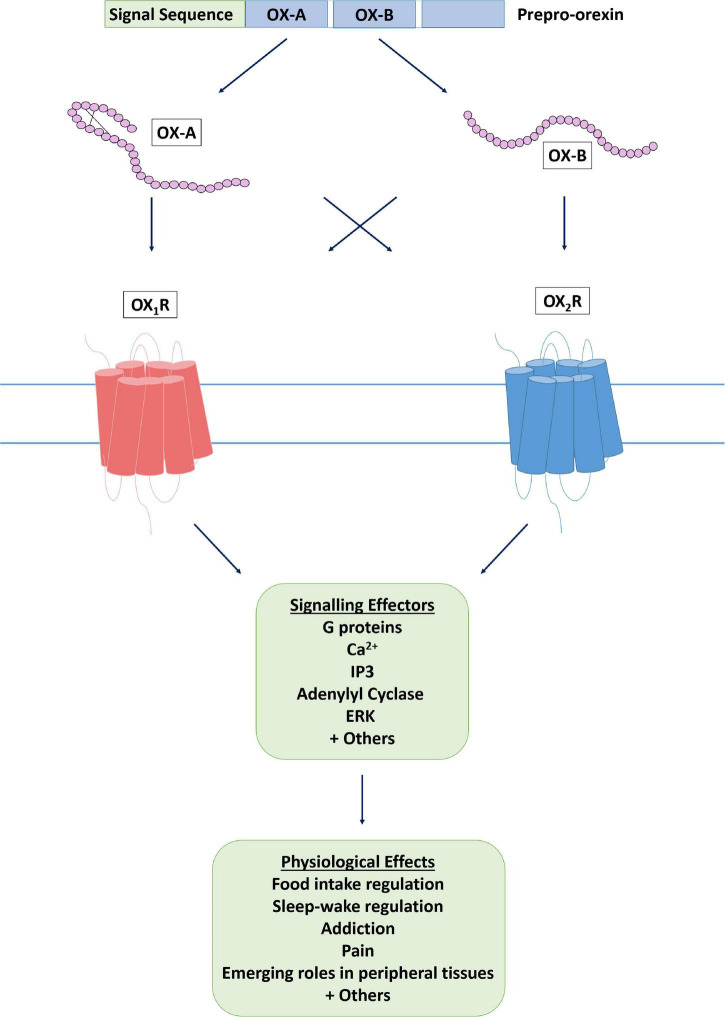
The orexin system. The orexin system consists of two endogenous peptide agonists, OX-A and OX-B, cleaved from the common precursor peptide prepro-orexin. Both agonists have affinity for the two orexin receptors, OX_1_R and OX_2_R, although it has been suggested that OX-B has some OX_2_R selectivity, whereas OX-A is not selective. Following agonist binding, an active conformation of the receptor is stabilized and effector proteins are recruited to induce a cellular response.

The orexin system was discovered in 1998 by two independent groups, De Lecea et al. (1998) and Sakurai et al. (1998). De Lecea et al. (1998) described an mRNA located within the hypothalamus that encodes for a peptide named prepro-hypocretin/orexin. Prepro-hypocretin/orexin, when cleaved, produces two highly conserved peptides termed hypocretin 1 and hypocretin 2. These peptides are also known as orexin-A (OX-A) and orexin-B (OX-B) respectively, and will be referred to using this terminology throughout this review. De Lecea et al. (1998) went on to show that Prepro-hypocretin/orexin was present specifically and exclusively in a limited population of cells in the dorsal and lateral hypothalamus (3,000–7,000 in rodents, up to 70,000 in humans), with immunoelectron microscopy studies indicating prepro-hypocretin/orexin associates with presynaptic vesicles. From these results, it was proposed that the OX-A and OX-B peptides were potential neurotransmitters, with their precursor’s localization in the lateral hypothalamus suggesting a role in energy homeostasis. Sakurai et al. (1998) also identified the presence of prepro-hypocretin/orexin mRNA within hypothalamic areas implicated in feeding behavior and demonstrated that administration of OX-A or OX-B into the lateral ventricles of rats resulted in increased food intake. However, Sakurai et al. (1998) first utilized reporter cell lines to de-orphanize G protein-coupled receptors (GPCRs), identifying the OX-A and OX-B peptides and the cognate OX_1_ and OX_2_ receptors (OX_1_R and OX_2_R, respectively) (Figure 1). This was part of a larger GPCR de-orphanization program (which included the orphan HFGAN72 sequence that corresponds to OX_1_R, allowing identification of two related expressed sequence tags that correspond to OX_2_R), conducted at the time in collaboration with Smith Kline Beecham, before their merger with Glaxo. Hence the two groups discovered the orexin system by two complementary approaches, identification of either the neuropeptides or the receptors first and then their respective receptor or neuropeptide counterparts in turn.

The discovery of OX-A and OX-B and the subsequent de-orphanization of OX_1_R and OX_2_R forms the basis of the orexin/hypocretin system as it is understood today. With regards to nomenclature, while hypocretin and orexin can be used interchangeably, it is most widely accepted to refer to related genes by the hypocretin name (in agreement with HUGO) while peptides and receptors are referred to by the orexin name (as recommended by IUPHAR, see Alexander et al. (2021), Coleman et al. (2021), so as to recognize the contributions of both discovery studies (De Lecea et al., 1998; Sakurai et al., 1998).

As previously mentioned, upon initial discovery, the orexin system was implicated in food intake regulation due to the prevalence of prepro-hypocretin/orexin and orexin receptors within the lateral hypothalamus – a brain region implicated in the regulation of food intake (De Lecea et al., 1998; Sakurai et al., 1998) – and subsequently the obese phenotype of orexin knockout mice. This localization evidence, combined with early observations that intracerebroventricular (ICV) administration of orexins resulted in an increase in feeding behavior in rats, heavily implicated the orexin system as a regulator of food intake (Sakurai et al., 1998; Edwards et al., 1999; Yamanaka et al., 2000). Further antagonist studies also supported this, with OX_1_R antagonists decreasing the orexigenic effect of OX-A administration (Haynes et al., 2000).

Further functional characterization and neuronal circuitry studies confirmed the orexin system as an important regulator of feeding behavior but also revealed orexin involvement in the regulation of other homeostatic mechanisms, in particular arousal and sleep-wake regulation (Peyron et al., 1998, 2000; Chemelli et al., 1999; Date et al., 1999; Edwards et al., 1999; Lin et al., 1999; Nambu et al., 1999; Yamanaka et al., 2000; Muroya et al., 2004; Sakurai and Mieda, 2011; Li and de Lecea, 2020). The focus of research into the orexin system took a marked pivot upon the observation that knockout of the prepro-hypocretin gene in mice produced a phenotype that exhibited striking similarities to human narcolepsy with cataplexy (Chemelli et al., 1999), also known as type 1 narcolepsy (NT1) (Mahoney et al., 2019). At the time, narcolepsy had no known cause and as such, this observation led to the rapid uptake of research into the orexin system’s role in the regulation of sleep (Lin et al., 1999; Scammell et al., 2000; Estabrooke et al., 2001; Saper et al., 2001; Sakurai, 2007; Tsujino and Sakurai, 2009). From this research came the discovery that the orexin system is a critical regulator of sleep-wake cycles, with the loss of orexin neurons (Thannickal et al., 2000, 2009; Hara et al., 2001; Honda et al., 2009) and the resulting loss of endogenous orexin (Nishino et al., 2000, 2001; Peyron et al., 2000; Ripley et al., 2001; Kanbayashi et al., 2002; Mignot et al., 2002) responsible for the rapid cycling between sleep and wake states, both during day and night, characteristic of human narcolepsy. The underlying cause of orexin neuron loss in human narcolepsy is still unknown, however autoimmune-related degenerative processes may play a role (Thannickal et al., 2000; Cvetkovic-Lopes et al., 2010; Lecendreux et al., 2017; Ramberger et al., 2017; Latorre et al., 2018; Luo et al., 2018; Bassetti et al., 2019; Lippert et al., 2019; Mahoney et al., 2019; Pedersen et al., 2019).

Additionally, the role of the orexin system in sleep-wake regulation was further validated when narcoleptic dogs were reported as having non-functional mutated OX_2_R (Lin et al., 1999; Hungs et al., 2001). This is particularly interesting, as similar receptor mutations have not been reported to cause narcolepsy in humans (Nishino et al., 2000, 2001; Peyron et al., 2000; Liblau et al., 2015). Indeed, NT1 is specifically linked to the loss of orexin producing cells in the hypothalamus and the resulting absence of measurable orexin in the cerebrospinal fluid of NT1 patients. Interestingly, orexin receptor mutant mice show different sleep phenotypes depending upon the receptor(s) mutated. OX_1_R knockout results in almost normal sleep-wake patterns, OX_2_R knockout results in a mild sleep phenotype with some sleep fractionation and very rare cataplectic events, whereas the double receptor knockout replicates the orexin peptide knockout (NT1) phenotype, with frequent cataplectic attacks, fast transitions from wake to REM sleep and sleep fragmentation (Willie et al., 2003; Mang et al., 2012; Hoyer and Jacobson, 2013; Jacobson et al., 2017, 2019). In particular, the sleep phenotype of the OX_2_R knockout mouse (Willie et al., 2003) did not replicate that of the orexin peptide knockout (Diniz Behn et al., 2010), nor that of the double receptor knockout, which both have fast transitions from wake to REM and cataplexy characteristic of NT1 in humans. The contributing role of OX_1_R to REM sleep modulation, was later confirmed in multiple studies showing that dual orexin receptor antagonists (DORAs) in rodents and humans over proportionally trigger REM sleep, whether during the active or inactive phase (Betschart et al., 2013; Hoyer and Jacobson, 2013; Hoyer et al., 2013). It has also become clear that DORAs have the ability to trigger cataplectic episodes in rodents, dogs and humans (Mahoney et al., 2020), and hence are contraindicated in patients suffering from NT1. This is also evident from studies combining OX_1_R and OX_2_R antagonists in rodents (Dugovic et al., 2009), where REM sleep is more affected than with an OX_2_R antagonist alone. Hence, there is contribution of both receptors to the phenotype displayed, however, the mechanistic basis for each receptors’ contribution is not yet understood, whether related to signaling and/or cellular/pathway crosstalk.

As these studies demonstrated that blockade/knockout of both receptors had a more pronounced effect on sleep than blockade/knockout of only one of the receptors, this led pharmaceutical and biotech companies to develop DORAs as a novel treatment for insomnia (Brisbare-Roch et al., 2007; Cox et al., 2010; Bettica et al., 2012; Coleman et al., 2012; Beuckmann et al., 2017; Berger et al., 2020; Boss et al., 2020). The critical role of OX_2_R was further evidenced in subsequent receptor antagonism/agonism studies performed in various transgenic mice which led to the alternate hypothesis that OX_2_R antagonism may produce more physiological sleep than DORAs (Mang et al., 2012; Betschart et al., 2013; Hoyer and Jacobson, 2013; Hoyer et al., 2013; Dugovic et al., 2014; Bonaventure et al., 2015; Clark et al., 2020). The DORA almorexant did not affect sleep-wake patterns in OX_2_R knockout mice, and was completely inactive in double receptor knockout mice, which incidentally confirmed that almorexant’s hypnotic effects were mediated exclusively by orexin receptors (Mang et al., 2012). Almorexant had major hypnotic effects in OX_1_R knockout mice, comparable to those seen in wildtype mice. However, a closer look at the polysomnography data suggested that almorexant’s effects on sleep were even more pronounced in the OX_1_R knockout (Mang et al., 2012). Along the same lines, OX-A produced no effect in double receptor knockout but had stimulatory effects in OX_1_R knockout mice, whereas it produced no obvious effects in OX_2_R knockout mice, emphasizing again a major role of the OX_2_R in wake-related activities (Mang et al., 2012). One unusual feature of a number of these DORAs is their very slow dissociation rates from the receptors, which result in sustained receptor occupancy and explain their long duration of action on top of rather long pharmacokinetic features (Mang et al., 2012; Callander et al., 2013; Farkas, 2013; Hoyer and Jacobson, 2013; Jacobson et al., 2014).

Since these initial studies revealing the orexin system’s role in critical homeostatic mechanisms, the system’s impact has been further expanded to include roles in addiction, pain, anxiety, panic, depression, binge eating and potentially post-traumatic stress disorder (PTSD) among others (Nakamura et al., 2000; Piccoli et al., 2012; Tsujino and Sakurai, 2013; Fitch et al., 2014; Li et al., 2014; Roh et al., 2014; Sakurai, 2014; Abbas et al., 2015; Bonaventure et al., 2015, 2017; Bonnavion et al., 2015; Soya et al., 2017; Steiner et al., 2018; Kaufmann et al., 2020). Additionally, a role for orexins and orexin receptors in peripheral tissues has also been suggested (Kirchgessner and Liu, 1999; Karteris et al., 2001, 2004, 2005; Mazzocchi et al., 2001; Randeva et al., 2001; Patel et al., 2018).

Mounting evidence has now demonstrated that classical G protein-dependent signaling at the plasma membrane is not the only way most GPCRs transduce signals. β-arrestin-dependent signaling (Smith and Rajagopal, 2016), signaling from GPCRs sequestered within subcellular compartments (Irannejad and von Zastrow, 2014; Thomsen et al., 2018) and bias toward specific signaling pathways due to ligand or cellular environment (Smith et al., 2018) have all been observed. Additionally, with the use of newly developed biosensors to probe G protein coupling to receptors [Wan et al., 2018; Laschet et al., 2019; Avet et al., 2020 (unpublished); Olsen et al., 2020], promiscuity in G protein coupling has been revealed to be more common than previously thought. Despite these shifts in dogma surrounding GPCR signaling, investigation of these concepts in relation to the orexin receptors has been very limited, with the understanding of their signaling properties mostly confined to the classical perspective. Here, the current understanding of orexin receptor interactions with direct effectors such as G proteins and β-arrestins, as well as the subsequent implications of these interactions for the signaling of the orexin system, will be discussed.

## Calcium and Intracellular Messengers

### Calcium Ion Movement

Ever since the orexin system’s discovery, activation of the orexin receptors has been strongly associated with a rise in intracellular Ca^2+^ (Sakurai et al., 1998). This is a hallmark of the receptors’ activation in all recombinant systems. Early studies aimed to elucidate the origin of the rise in cytoplasmic Ca^2+^ in order to give insight into the cellular mechanisms and transduction cascades involved in orexin receptor signaling.

Van Den Pol et al. (1998) used the fluorescent free Ca^2+^ indicator Fura-2 to perform imaging on hypothalamic neurons to characterize Ca^2+^ elevations upon stimulation with orexins. They observed that depletion of intracellular Ca^2+^ stores by pre-treatment with the endoplasmic reticulum Ca^2+^ ATPase inhibitor thapsigargin had no significant effect on the orexin-induced response. However, stimulation of cells in the presence of a chelating agent (EGTA) abolished the rise in intracellular Ca^2+^ concentration in response to stimulation with orexins.

Smart et al. (1999) subsequently utilized a Fluorescence Imaging Plate Reader (FLIPR) along with CHO cells transfected with OX_1_R or OX_2_R to characterize the rise in intracellular Ca^2+^. When OX_1_R was stimulated with 1 μM OX-A, a rapid peak in intracellular Ca^2+^ followed by a slow decline in the elevated Ca^2+^ level was observed. It is noted that similar results were observed for OX-B at OX_2_R, however these data were not shown. In contrast to Van Den Pol et al. (1998)’s findings, Smart et al. (1999) observed a rise in intracellular Ca^2+^ in the absence of extracellular Ca^2+^, however also observed that the Ca^2+^ response became more transient. Depletion of intracellular Ca^2+^ stores abolished the OX_1_R response to OX-A and OX-B. In addition, this study demonstrated that inhibition of phospholipase C (PLC) by U73122 abolished the Ca^2+^ response. These results were suggested to indicate that the orexins stimulate the release of intracellular Ca^2+^ stores for the rapid peak response as well as extracellular Ca^2+^ influx for the prolonged response, resulting in the typical biphasic pattern.

Lund et al. (2000) however, support extracellular Ca^2+^ influx as the primary mechanism of intracellular Ca^2+^ increase as a result of OX_1_R stimulation. Lund et al. (2000) demonstrate OX_1_R-mediated activation of cation channels and the resultant rise in intracellular Ca^2+^ acting synergistically with Gα_*q*_ activation to increase PLC activation. These results suggest that inositol trisphosphate (IP_3_)-mediated Ca^2+^ release from intracellular stores is secondary and dependent upon extracellular Ca^2+^ influx for OX_1_R. This finding is reiterated in Larsson et al. (2005) and Johansson et al. (2007), who also implicate transient receptor potential cation channels (TRPCs) as mediating Ca^2+^ influx. Interestingly, these studies demonstrate the Ca^2+^ influx dependency of the response decreases at high concentrations of OX-A (0.1 μM and above), however the physiological relevance of this is unclear.

From these initial studies, subsequent works aimed to further elucidate the mechanisms underlying the robust Ca^2+^ response characteristic of orexin receptor activation. Studies in recombinant cells largely support a primary role for extracellular Ca^2+^ influx (Peltonen et al., 2009; Putula et al., 2014; Wang et al., 2014; Nagahara et al., 2015), however, divergences in function have been described between these recombinant systems and measurements in neurons. Indeed, there is conflicting evidence between neuronal studies (Van Den Pol et al., 1998; Uramura et al., 2001; Kohlmeier et al., 2004, 2008; Muroya et al., 2004; Kukkonen and Leonard, 2014). In summary, it appears that the orexin-induced intracellular Ca^2+^ increase is primarily a result of extracellular Ca^2+^ influx, with a small contribution from intracellular Ca^2+^ release, with cell- and tissue-specific effects also likely.

### Downstream Signaling Interactions

Orexin receptor activation has been documented to result in an enormously varied set of downstream interactions, involving many key cascades within the signaling milieu. Studies investigating responses downstream of G protein activation indicate the involvement of multiple concurrent signaling mechanisms. The activation of effectors classically thought of as directly downstream of G protein activation are often used as indicators of specific G protein activity, despite the two not always being directly correlated. The activation of PLC, the production of diacylglycerol (DAG) and IP_3_ and an increase in intracellular Ca^2+^ have been widely characterized upon stimulation of orexin receptors. The activation of adenylate cyclase and production of cyclic adenosine monophosphate (cAMP) have also been characterized in some studies (Randeva et al., 2001; Tang et al., 2008; Kukkonen, 2016a,b).

Further downstream, activation of extracellular signal-regulated kinases (ERKs) (Ammoun et al., 2006a), phospholipase A_2_ (PLA_2_) (Turunen et al., 2012), phospholipase D (PLD) (Jäntti et al., 2012) and p38 mitogen-activated protein kinases (Ammoun et al., 2006b) among others (Ammoun et al., 2006a; Turunen et al., 2012) have been reported, once again highlighting the diverse signaling capabilities of the orexin system, the functional consequences of which are still being elucidated. To date, much of this research has been conducted within recombinant cell environments with a bias toward studying OX-A-induced OX_1_R function. Therefore an important part of future research will be examining these aspects of orexin receptor signaling within primary cell models and native tissue, as well as identifying how much of the OX-A-induced OX_1_R-derived signaling patterns can be applied to OX_2_R- and OX-B-induced signaling. Kukkonen (2016b) began to address this, characterizing the signaling effectors activated by OX_2_R in CHO cells and comparing the profile to that of OX_1_R. They observed the involvement of the same effectors described for OX_1_R with minor differences in potency for some signaling pathways. However, they did not investigate the effect of OX-B-induced stimulation and as such, any ligand-dependent effects at OX_2_R on the signaling pathways investigated remain to be characterized in such detail.

Further information on the downstream effectors and signaling cascades implicated in orexin function is summarized in Kukkonen and Leonard (2014) and Leonard and Kukkonen (2014).

## G Proteins

As a result of receptor activation leading to increased intracellular Ca^2+^ levels and PLC activation, the orexin receptors were initially thought to couple to Gα_*q*_ with subsequent PLC-IP_3_ signaling (Sakurai et al., 1998; Lund et al., 2000; Holmqvist et al., 2002). Further research expanded the G protein-coupling partners of the orexin receptors, with evidence of varying degrees of coupling to Gα_*q*_, Gα_*i/o*_ and Gα_*s*_, depending upon the experimental conditions. Given this variation in the reported coupled G proteins, there is uncertainty around the relevance of these reports to endogenous function as the literature contains multiple accounts of conflicting evidence. A number of these discrepancies can be attributed, at least in part, to the varied cell and tissue conditions under which orexin signaling has been studied. This possible tissue- and cell-dependent variation in the G protein coupling indicates that the effector proteins may be influenced by different environmental conditions (Randeva et al., 2001; Caillol et al., 2003; Woldan-Tambor et al., 2011). Other factors may also contribute to these perceived differences, including the stimulating ligand used, ligand concentration and experimental method. Of particular note is the abundance of G protein coupling data that has been inferred based upon assays that detect the activation of downstream messengers of G proteins. This has been historically necessary due to the lack of techniques to study G protein coupling directly. However, given the well-characterized promiscuity of the orexin receptors’ G protein coupling [Avet et al., 2020 (unpublished)], and the possibility for cross-regulation between classical signaling cascades (Houslay and Kolch, 2000; Halls and Cooper, 2011), caution must be used when applying these data to determine G protein coupling. Here, we will look in-depth at the evidence for G protein coupling to each of the orexin receptors.

### G Protein Coupling to Both Receptors

In their discovery study, Sakurai et al. (1998) suggested that the orexin receptors couple to Gα_*q*_ due to the generation of OX-A or OX-B concentration-dependent increases in intracellular Ca^2+^ in CHO cells stably transfected with OX_1_R or OX_2_R. Van Den Pol et al. (1998) subsequently gave indirect evidence for coupling to Gα_*q*_, as inhibition of PKC using the inhibitor bisindolylmaleimide abolished orexin-induced intracellular Ca^2+^ increase. However, which specific orexin receptor was mediating this effect in the hypothalamic neurons was not suggested or investigated.

Randeva et al. (2001) detected OX-A-induced activation of Gα_*q*_, Gα_*s*_, and Gα_*i*_ in human adult adrenal gland membrane preparations using a GTP-azidoanilide-labeling and subsequent immunoprecipitation technique. Subsequent functional analysis showed a concentration-dependent increase in both cAMP and IP_3_ production upon treatment with OX-A. These findings are in agreement with previous data detailing increased cAMP levels in adrenal cells upon stimulation with orexins (Malendowicz et al., 1999; Mazzocchi et al., 2001). Randeva et al. (2001) also characterized the receptors present within the adrenal tissue. Using RT-PCR, the expression of OX_2_R, but not OX_1_R, was observed and this was confirmed using various additional methods, as was the presence of prepro-orexin. The orexin expression pattern in adrenal tissue is contested however, as discussed in the “*Signaling in Peripheral Cell and Tissue Environments”* section below.

Zhu et al. (2003) utilized neuronal hybrid cells (BIM cells) stably expressing OX_1_R-EGFP or OX_2_R-EGFP to measure the effect of orexin stimulation on cAMP and intracellular Ca^2+^ levels. From the trends observed in these pathways, Zhu et al. (2003) suggest coupling of both receptors to Gα_*q*_, OX_2_R but not OX_1_R coupling to Gα_*i*_, and coupling of neither receptor to Gα_*s*_. However, as this study only investigated effectors downstream of G protein coupling, and had limited use of pathway modulators [such as pertussis toxin (PTX) and cholera toxin (CTX)], these suggestions should be treated with caution. The lack of data on the effect of OX-B stimulation on cAMP in this study is also of note. A bias toward studying OX-A-induced responses is present within the literature, with markedly less data available on the effects of OX-B. This is unfortunate, as ligand-specific effects have been reported for the orexin system and therefore important signaling events have potentially been missed due to the omission of OX-B in such studies. Ligand-specific effects that have been elucidated are discussed in detail in the “*Endogenous Ligands”* section below.

### OX_1_R G Protein Coupling

Multiple studies followed that aimed to investigate the coupling of OX_1_R to G proteins and the associated signaling cascades. Holmqvist et al. (2005) utilized CHO cells stably expressing OX_1_R to study the response to orexins of multiple downstream G protein effectors. CTX (which constitutively activates Gα_*s*_) and PTX were used to probe for potential G protein mediation with both positive and negative regulation of cAMP production observed. Robust and concentration-dependent intracellular Ca^2+^ elevation and inositol phosphate production was also observed. From these results, the authors suggest that in CHO cells, OX_1_R couples to a minimum of three G proteins: Gα_*i/o*_, Gα_*s*_, and Gα_*q*_.

Magga et al. (2006) used immunoprecipitation of C-terminally FLAG-tagged OX_1_R and subsequent immunoblotting with Gα subunit-specific antibodies to investigate the G protein coupling partners of OX_1_R in HEK293 cells. Gα_*q*_ showed the strongest signal for co-immunoprecipitation with OX_1_R-FLAG and this interaction was detected in all experiments. Co-immunoprecipitation was also observed with Gα_*i*_ and Gα_*s*_ but this was not detected in all experiments (6/11 and 2/3 independent experiments respectively) with no detection of Gα_*o*_ (0/5). Subsequent functional analysis showed stimulation of HEK293 cells stably expressing OX_1_R-FLAG with OX-A resulting in no change to cAMP levels from basal or when pre-treated with forskolin. From this study, it is not clear if OX_1_R couples to Gα_*s*_ or Gα_*i*_ in HEK293 cells. While the co-immunoprecipitation data indicate weak coupling may be present, the functional data were unable to support this.

Woldan-Tambor et al. (2011) observed robust cAMP production in rat cerebral cortex astrocyte cultures upon stimulation with OX-A. This effect was not seen upon treatment with the moderately OX_2_R selective agonist [Ala^11^,D-Leu^15^]-OX-B and was partially inhibited by an OX_1_R-selective antagonist (SB 40812), but not by the OX_2_R-selective antagonist (TCS OX2 29).

### OX_2_R G Protein Coupling

Research focused specifically on OX_2_R came somewhat later than that on OX_1_R. Using dominant negative G proteins in HEK293 cells stably expressing OX_2_R, Tang et al. (2008) observed Gα_*q*_, Gα_*s*_ and Gα_*i*_ involvement in orexin-induced ERK activation. Even though involvement of all three G proteins in downstream effector activation was detected, there was evidence for ligand-specific bias in the activated G proteins and cascades. This is discussed in detail in the “*Endogenous Ligands*” section, along with potential limitations of the methodologies used in the study.

These findings were in stark contrast to the findings of Urbańska et al. (2012) in primary cultures of rat cortical neurons which showed no effect of orexin stimulation on cAMP levels, suggesting no direct activation via Gα_*s*_ or any indirect activation, e.g., via PLC. They did, however, observe inhibition of forskolin-induced cAMP accumulation with orexin stimulation, which was completely reversed by PTX treatment, as well as inhibited by an OX_2_R-selective antagonist (TCS OX2 29) but not an OX_1_R-selective antagonist (SB 408124). These results suggest OX_2_R-mediated activation of Gα_*i*_ is observed upon stimulation with orexins in this particular cell model.

Kukkonen (2016a) revisited the coupling partners of both OX_1_R and OX_2_R in CHO cells by investigating the effect of G protein inhibitors on downstream signaling effectors. It was observed that for both receptors, Gα_*q*_ was responsible for the vast majority of responses, including intracellular Ca^2+^ accumulation, which was abolished with UBO-QIC (Gα_*q*_ inhibitor) treatment. Their results also gave evidence for Gα_*s*_-mediated adenylate cyclase activity with OX-A stimulation at both receptors. Additionally, Kukkonen (2016b) suggested that OX_2_R has weaker coupling to Gα_*s*_ relative to OX_1_R in CHO cells, as only weak adenylate cyclase activity could be detected upon stimulation with OX-A. Interestingly, UBO-QIC and PTX were found to reduce and abolish, respectively, the inhibitory effect of OX-A stimulation on adenylate cyclase for OX_1_R and OX_2_R, indicating this effect that has classically been attributed to Gα_*i*_ may also be partially mediated by Gα_*q*_. It is noteworthy that while these studies provide an excellent comparison of OX_1_R and OX_2_R G protein coupling and downstream signaling, no investigation with OX-B stimulation was pursued. Given that biased agonism of OX-A and OX-B to specific G protein signaling cascades has been reported previously (Tang et al., 2008), this would be an interesting aspect to investigate further.

More recently, Baker and Proudman (2021) reported a biphasic cAMP response to OX-A and OX-B in CHO cells expressing human OX_2_R but not in OX_1_R expressing cells. The inhibitory, but not the stimulatory component, was PTX sensitive. Both OX-A and OX-B were highly potent activators of Ca^2+^ release, inositol phosphate (IP) accumulation and ERK1/2 activity, with moderate or significant selectivity for OX-B. Interestingly, in the same study compound C (Nagahara et al., 2015) was potent and OX_2_R selective in the latter three assays, but had no effect on cAMP accumulation, suggesting biased signaling. Furthermore, the potency values of OX-B varied between OX_2_R-mediated Ca^2+^ release, IP accumulation, ERK1/2 activity and cAMP inhibition and activation (pEC_50_ = 10.36, 9.84, 11.08, 9.68, and 7.85). Similar differences were observed for OX-A in the same assays (pEC_50_ = 9.72, 9.28, 10.47, 9.54, and 7.72). The potency variations of OX-A and OX-B at the OX_1_R were less marked. Altogether, these data would suggest that natural and synthetic agonists can display pathway selectivity at human OX_2_R, with a range of potency and/or apparent efficacy values that are signaling dependent. Similar although less marked differences were noticed at OX_1_R, except that cAMP was not affected by any of the ligands tested.

## Signaling in Peripheral Cell and Tissue Environments

Studies often characterize the presence of orexins and orexin receptors within cells or tissue by utilizing techniques such as RT-PCR, western blot and fluorescence *in situ* hybridization (FISH). In some cases, a predominant receptor subtype can be identified, allowing for signaling characteristics to be attributed as predominantly OX_1_R- or OX_2_R-mediated. However, when both receptors are detected, dissecting receptor-specific signaling becomes more difficult and requires the use of receptor-specific antagonists or other tools. While orexin receptors can be located within the same areas of the CNS, they most often show distinct, complementary distribution (Marcus et al., 2001; Sakurai and Mieda, 2011). As such, research using CNS-derived cells and tissues rarely encounter this issue, with signaling observations often attributable to the one present orexin receptor subtype. In contrast, expression of both receptors appears to be more common within the periphery, and as such, techniques to investigate the contribution of each receptor are necessary in these studies.

In addition, there are conflicting data on the expression of orexin receptors within peripheral environments, making analysis of results in these studies difficult (Heinonen et al., 2008; Kukkonen, 2013). Perhaps the most prominent example of this is in adrenal gland tissue and cells, in which some studies report only OX_2_R expression (Karteris et al., 2001; Randeva et al., 2001) while others report expression of both OX_1_R and OX_2_R (Lopez et al., 1999; Spinazzi et al., 2005).

These conflicting reports may be attributable to both lack of binding to orexin receptors and lack of specificity of anti-orexin receptor peptide antibodies (Kukkonen, 2013), although inter-species and intra-organ (e.g., adrenal cortex vs. medulla) differences may also contribute (Heinonen et al., 2008). As such, while signaling behaviors are sometimes attributed to specific receptors, this analysis should be approached with caution.

### Adrenal Tissue

One of the earliest studies to investigate peripheral orexin signaling and G protein coupling was the previously mentioned study by Randeva et al. (2001) which gave evidence for OX_2_R coupling to Gα_*q*_, Gα_*s*_, and Gα_*i*_, but not Gα_*o*_ in human adult adrenal tissue. A similar study in human fetal adrenal tissue supported the finding of OX_2_R, but not OX_1_R expression within human adrenal glands. However, interestingly, upon stimulation with OX-A, coupling of Gα_*s*_ and Gα_*i*_ but not Gα_*q*_ was detected by GTP-azidoanilide labeling (Karteris et al., 2001). Why this difference between fetal and adult coupling was observed is unclear and warrants further investigation, although it is not uncommon to observe differences in protein expression between fetal and adult tissues. Also of note, both these studies investigated OX-A-induced responses only, as OX-B was not tested.

Contrasting results subsequently gave evidence for coupling of Gα_*q*_, Gα_*s*_, Gα_*i*_ as well as Gα_*o*_ in rat adrenal cortex and also the hypothalamus (Karteris et al., 2005), however, this may represent a species-specific difference. Remarkably, this study also showed changes to the G protein activation profile upon OX-A stimulation following 24 h of food deprivation. Subsequent work to further interrogate this change in G protein activation would assist in understanding the implications of this finding, for example, analysis of the contribution of each receptor. However, this study serves as yet another example of the potentially fluid and highly variable nature of orexin signaling.

Ramanjaneya et al. (2008) investigated orexin signaling in the H295R adrenocortical cell line. Steroidogenic acute regulatory protein (StAR) is a transporter protein important to acute steroid synthesis. Ramanjaneya et al. (2008) demonstrated that both orexin ligands upregulate expression of StAR in H295R cells. Using dominant negative G proteins, which it should be noted can have issues of non-specificity (Kukkonen, 2004), they subsequently suggested the involvement of Gα_*q*_, Gα_*s*_, and Gα_*i*_ in OX-A-induced StAR expression, but only Gα_*q*_ and Gα_*s*_ involvement in OX-B-induced StAR expression. An OX_1_R-selective antagonist was used to determine the role of each orexin receptor in these responses, with OX_1_R found to be mediating OX-A-induced coupling, and both receptors, but predominantly OX_1_R, mediating the OX-B-induced response. In a second study, Ramanjaneya et al. (2009) investigated the regulation of mitogen-activated protein kinase (MAPK) activation by the orexin system, showing distinct G protein coupling to ERK/MAPK cascade regulation for both ligands. They suggested Gα_*q*_, Gα_*s*_, and Gα_*i*_ involvement that was predominantly OX_1_R-mediated, with minor input from OX_2_R for both ligands’ effects. Although these studies did not investigate any possible activation or involvement of Gα_*o*_ within this cell model, they regardless give insight into the potential complexity of orexin-G protein coupling within adrenal tissue.

Orexin receptors have been detected within many peripheral tissues (Johren et al., 2001; Heinonen et al., 2008), however G protein coupling of the orexin system within these tissues has not been widely investigated, with the most in-depth analysis found in the adrenal glands as discussed above. Future research in a range of peripheral tissues may shed light on the enigmatic orexin G protein coupling systems. Furthermore, an area of orexin research that has been reinvigorated by peripheral tissue studies pertains to the roles of the two orexin ligands and the potential differences in their signaling and function. Further discussion of this can be found in the “*Endogenous Ligands”* section.

## Non-G Protein Modulation

While much attention has been given to G protein activation and the subsequent signaling cascades, the role of other effector proteins in orexin pharmacology has been less thoroughly interrogated. Important receptor accessory proteins, such as arrestins, have the ability to impact signaling behavior, and therefore understanding these interactions could reveal important insights into the function of orexin receptors.

### β-Arrestins

Both orexin receptors have been shown to interact robustly with β-arrestins. Along with their classical role in signal cessation and receptor internalization, β-arrestins also scaffold secondary signaling platforms (Lefkowitz and Shenoy, 2005; DeWire et al., 2007).

Evidence that β-arrestin2 plays a role in orexin signaling through ERK pathways is given by Milasta et al. (2005). Notably, ERK1/2 phosphorylation was reduced and the time-scale of ERK1/2 phosphorylation shortened with a mutant OX_1_R that reduced agonist-induced β-arrestin2 recruitment. This was despite no significant changes to the induction of increased intracellular Ca^2+^, indicating these changes do not result from general reduced signaling capacity. Additionally, while β-arrestin2-dependent internalization of the mutated receptor was minimally impacted, colocalization of OX_1_R and βarrestin-2 within endosomal compartments following internalization was not observed with the mutant receptor, in contrast to the wildtype OX_1_R. This study did not investigate whether the same patterns could be observed with OX_2_R or OX-B treatment, however subsequent studies suggest OX_1_R and OX_2_R have distinct interaction profiles with β-arrestin2.

**FIGURE 2:**
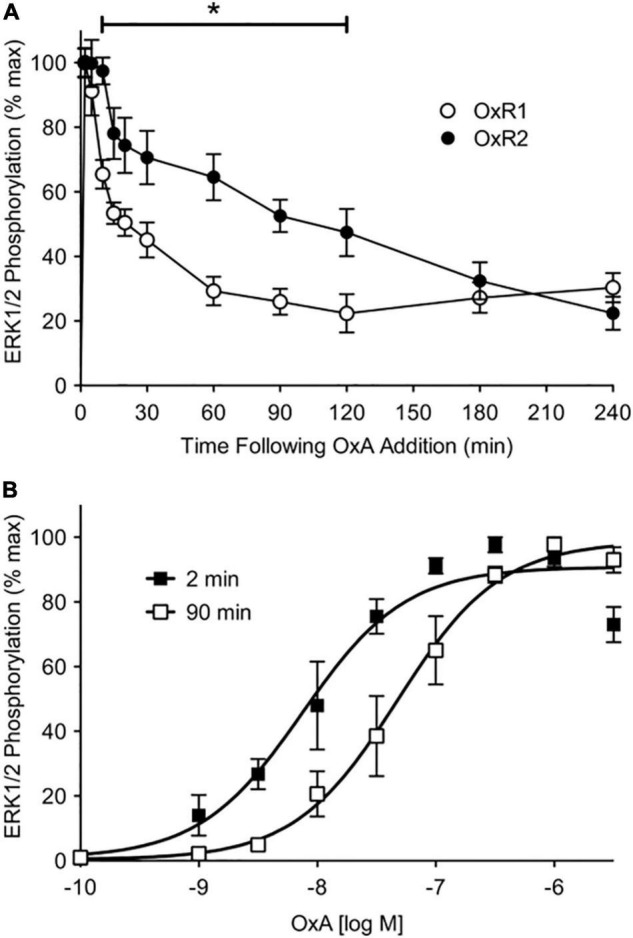
ERK1/2 phosphorylation data for OX_1_R (OxR_1_) and OX2R (OxR_2_) stably transfected in HEK293 cells. **(A)** Stably transfected HEK293 cells were treated with OX-A (OxA) and measured over a 4-h period. Data were normalized to time-matched vehicle treatments and are expressed as a percentage of the maximal response induced at 2 min post-agonist treatment. Data are expressed as mean ± SE of four independent experiments. *p < 0.05 between OX_1_R and OX_2_R from 10 to 120 min post-agonist stimulation. **(B)** Concentration-response data were collected at 2- and 90-min post OX-A treatment of OX_2_R-expressing cells. Data are expressed as a percentage of the maximal response induced at the time point (mean ± S.E. of four independent experiments). Reproduced from Dalrymple et al. (2011) under a Creative Commons Attribution 4.0 International License. Full terms provided at https://creativecommons.org/licenses/by/4.0/.

In COS-7 cells, Dalrymple et al. (2011) observed β-arrestins to be recruited with a higher potency to OX_2_R compared to OX_1_R in response to OX-A. Using a kinetic BRET assay, OX_1_R underwent relatively transient interactions with arrestin-ubiquitin complexes compared to OX_2_R that showed more stable and prolonged interactions. Additionally, ERK1/2 phosphorylation in response to OX-A stimulation was similar for both receptors in the short term (up to 10 min post-stimulation), however, OX_1_R exhibited lower levels of sustained ERK1/2 phosphorylation compared to OX_2_R (Figure 2). Dalrymple et al. (2011) suggested β-arrestins may form a more stable secondary signaling platform with OX_2_R than with OX_1_R, indicating a mechanism that may play a role in the diverging functional profiles of the two receptors.

Possible structural determinants of the orexin-β-arrestin interaction profiles were investigated by Jaeger et al. (2014). Using site-specific mutagenesis to isolate the contribution of serine and threonine residues in the C-terminal tail of OX_2_R, they showed that OX_2_R has two C-terminal serine/threonine clusters that are phosphorylated by GRKs, creating a strong affinity for the receptor with β-arrestin. This is in contrast to OX_1_R which has only one C-terminal serine/threonine cluster important for GRK phosphorylation, resulting in the less stable interactions reported by Dalrymple et al. (2011). This difference in C-terminal phosphorylation sites is also suggested to be the structural determinant underpinning the slower recycling rate of OX_2_R observed by Dalrymple et al. (2011), with OX_2_R requiring more significant dephosphorylation post-internalization to dissociate from β-arrestin, resulting in the receptor remaining within the cell for longer. These results were produced using only OX-A stimulation. Whether different structural determinants may be involved in OX-B-induced β-arrestin2 interactions is unknown. Regardless, these foundational studies give insight into the role of β-arrestin in orexin signaling and demonstrate the potential importance of non-G protein interactions.

With the exception of arrestins, research investigating the potential role of accessory proteins in the control of orexin receptor signaling is sparse. Further in-depth investigation into the role of such proteins could uncover further modulators of orexin signaling. This could ultimately aid in explaining the complex signaling and functional characteristics described for the orexin system.

### Heteromerization

Heteromerization has been suggested to play a functional role in the orexin system, with particular focus on heteromerization of orexin receptors with opioid and cannabinoid receptors (Thompson et al., 2017). However, it is of note when considering these reports that these studies rely heavily on recombinant expression, which is prone to overexpression artifacts. Reported functional interactions between orexin receptors and other GPCRs are listed in Table 1.

**TABLE 1:** Select reported interactions between orexin receptors and other GPCRs in native and recombinant environments.

Orexin receptor	Interacting receptor	Reported findings
OX_1_R	Kappa opioid receptor (κOR)	OX_1_R and κOR were suggested to co-express in rat hippocampal neurons. In HEK293 cells, colocalization/heteromerization lead to enhancement of Gα_*s*_, PKA and cAMP signaling (Chen et al., 2015). Another study, however, suggested the interaction occurs at the level of downstream signaling pathways (Robinson and McDonald, 2015).
OX_1_R	Corticotropin-releasing factor receptor 1 (CRF_1_R)	In HEK293 cells and in rat ventral tegmental area slices, CRF_1_R-OX_1_R heteromers caused negative crosstalk of OX-A and CRF signaling. The heteromer also complexes with the cocaine target σ1 receptor. This promotes long-term disruption of the OX-A and CRF negative crosstalk that modulates dendritic dopamine release (Navarro et al., 2015).
OX_1_R	Corticotropin-releasing factor receptor 2 (CRF_2_R)	In HEK293 cells, CRF_2_R-OX_1_R heteromers cross-antagonized one another; this was potentiated by amphetamine and potentially involved formation of a higher order complex with σ1 and σ2 receptors. In rat ventral tegmental area slices, amphetamine potentiated OX-A-induced dopamine and glutamate release, which was blocked by CRF_2_R antagonism (Navarro et al., 2019).
OX_1_R	Growth hormone secretagogue receptor 1a (GHS-R1a; ghrelin receptor)	In HEK293 cells, GHS-R1a-OX_1_R heteromers cross-antagonized one another’s signaling, while formation of a trimeric complex with the leptin receptor abolished this antagonism. In primary cultures of hypothalamic neurons, agonist responses resembled those mediated by the trimeric rather than the dimeric complexes (Medrano et al., 2018).
OX_1_R	Cholecystokinin A receptor (CCK_1_)	In HEK293 cells, OX_1_R and CCK_1_ formed heteromers. Dual receptor activation reduced G protein signaling and migration, but not β-arrestin interactions, compared to single receptor activation (Bai et al., 2017).
OX_2_R	5-hydroxytryptamine 1A receptor (5-HT_1*A*_)	OX_2_R and 5-HT_1*A*_ are suggested to colocalize in rat hippocampus slices and in the cell membrane of HEK293T cells. Receptor co-expression lead to increased cAMP and Ca^2+^ signaling, and decreased ERK signaling (Wang et al., 2019).
OX_1_R OX_2_R	Cannabinoid receptor type 1 (CB_1_)	In Flp-In T-REx 293 cells, OX_1_R and CB_1_ formed heteromers. OX-A induced internalization of CB_1_, with increased potency compared to OX-A-induced internalization of OX_1_R (Ward et al., 2011). BRET evidence for formation of CB_1_-OX_1_R, CB_1_-OX_2_R and OX_1_R-OX_2_R heteromers (Jäntti et al., 2014).

The effects of orexin receptor heteromerization include cross-activation and inhibition, as well as novel signaling mechanisms that are different from those of the monomeric receptor. An example of this is seen with the kappa opioid receptor (κOR)-OX_1_R heteromer, in which signaling through Gα_*s*_ is observed in response to OX-A or dynorphin A, with a reduction in Gα_*i*_ and Gα_*q*_ signaling through κOR and OX_1_R, respectively, upon heteromerization (Chen et al., 2015). Heteromerization resulting in novel signaling is strong evidence of a functional interaction between the two receptors that may result in physiologically relevant applications. Given the wide range of reported heteromers from the orexin system, the formation of higher order receptor oligomers is likely to be an important aspect of the orexin system’s complex *in vivo* functions, and may underlie some of the reported signaling variation in different cell and tissue types. Further research is needed to determine the extent of the influence of heteromerization on these factors, as well as further characterize the novel signaling properties of specific orexin heteromers.

## Ligands

### Endogenous Ligands

The orexin system has two known endogenous ligands, OX-A and OX-B, both resulting from a common precursor, prepro-orexin. OX-A is a 33 amino acid peptide with two intrachain disulfide bonds, while OX-B is a 28 amino acid linear peptide. Both orexins have a C-terminal amide group (Sakurai et al., 1998). In their first report of hypocretin/orexin peptides, De Lecea et al. (1998) reported the fast neuroexcitatory effects of the native OX-B (1 μM) in various rat hypothalamic neuronal cultures and the absence of effects of OX-B in four different hippocampal dentate granule neuronal cultures. In these early studies, OX-A activity was not tested.

In CHO and HEK293 cells, OX_1_R shows preferential binding of OX-A over OX-B while at OX_2_R, OX-A and OX-B have similar affinities in radioligand binding experiments (Sakurai et al., 1998). Similar trends are reflected in the potency of the ligands at the two receptors, with OX-A and OX-B showing equivocal potency at OX_2_R in IP production assays while OX-B shows significantly lower potency at OX_1_R (Holmqvist et al., 2002; Dalrymple et al., 2011; Kocan et al., 2011). Despite these characterizations, many nuanced ligand-specific effects have been reported, the roles of which remain to be elucidated. In other words, the putative receptor selectivity of OX-B needs to be re-considered, depending on cellular environment and/or transduction mechanism considered. The data reported by different groups in a variety of cells (whether collected from recombinant systems or from primary cultures) and/or assays, demonstrate variations in apparent affinity, potency and to some extent efficacy (see above, e.g., Section “OX_2_R G protein coupling”). Therefore, the purported “selectivities” for endogenous and synthetic ligands, especially agonists, show marked variations and the use of “selective” when describing such ligands must be taken with caution. An obvious consequence of this, is that effects mediated by endogenous orexin receptors need to be characterized using adequate pharmacological tools, preferably by measuring rank orders of potency of several antagonists with various degrees of selectivity, rather than the limited use of poorly selective agonists.

**TABLE 2:** Described orexin ligands.

Name	Selectivity	Peptide or Non-peptide	Chemical name/Peptide sequence
**Antagonists**			
SB-334867	OX_1_R selective antagonist	Non-peptide	1-(2-methylbenzoxazol-6-yl)-3-[1,5]naphthyridin-4-yl urea
SB-408124	OX_1_R selective antagonist	Non-peptide	1-(6,8-difluoro-2-methylquinolin-4-yl)-3-(4-dimethylaminophenyl)urea
SB-410220	OX_1_R selective antagonist	Non-peptide	1-(5,8-difluoroquinolin-4-yl)-3-(4-dimethylaminophenyl)urea
SB-674042	OX_1_R selective antagonist	Non-peptide	[5-(2-Fluorophenyl)-2-methyl-4-thiazolyl][2(S)-2-[(5-phenyl-1,3,4-oxadiazol-2-yl)methyl-1-pyrrolidinyl]methanone
ACT-335827	OX_1_R selective antagonist	Non-peptide	(2R)-2-[(1S)-1-(3,4-Dimethoxybenzyl)-6,7-dimethoxy-3,4-dihydro-2(1H)-isoquinolinyl]-*N*-isopropyl-2-phenylacetamide
ACT-539313	OX_1_R selective antagonist	Non-peptide	((4-methyl-2-[1,2,3]triazol-2-yl-phenyl)-[(R)- 3-(3-[1,2,3]triazol-2-yl-benzyl)-morpholin-4-yl]-methanone) (Kaufmann et al., 2020)
Nagase 71	OX_1_R selective antagonist	Non-peptide	(E)-*N*-((4R,4aS,7R,7aR,12bS)-3-{[2-(Dimethylamino)phenyl]sulfonyl}-4a-hydroxy-9-methoxy-2,3,4,4a,5,6,7,7a-octahydro-1H-4,12-methanobenzofuro[3,2-e]isoquinolin-7-yl)-N-methyl-3-(pyridin-2-yl)acrylamide (Nagase et al., 2017)
GSK-1059865	OX_1_R selective antagonist	Non-peptide	[5-bromo-N-({1-[(3-fluoro-2-methoxyphenyl)carbonyl]-5-methylpiperidin-2-yl}methyl)pyridin-2-amine]
JNJ-54717793	OX_1_R selective antagonist	Non-peptide	(1S,2R,4R)-7-([(3-fluoro-2-pyrimidin-2-ylphenyl)carbonyl]-*N*-[5-(trifluoromethyl)pyrazin-2-yl]-7-azabicyclo[2.2.1]heptan-2-amine) (Bonaventure et al., 2017)
TCS-OX2-29	OX_2_R selective antagonist	Non-peptide	(2S)-1-(6,7-dimethoxy-3,4-dihydro-1H-isoquinolin-2-yl)-3,3-dimethyl-2-(pyridin-4-ylmethylamino)butan-1-one
EMPA	OX_2_R selective antagonist	Non-peptide	*N*-ethyl-2-[(6-methoxypyridin-3-yl)-(2-methylphenyl)sulfonylamino]-N-(pyridin-3- ylmethyl)acetamide
JNJ-10397049	OX_2_R selective antagonist	Non-peptide	*N*-(2,4-dibromophenyl)-*N′*-[(4S,5S)-2,2-dimethyl-4-phenyl-1,3-dioxan-5-yl]-urea
JNJ-42847922 (Seltorexant)	OX_2_R selective antagonist	Non-peptide	([5-(4,6-dimethyl-pyrimidin-2-yl)-hexahydro-pyrrolo[3,4-c]pyrrol-2-yl]-(2-fluoro-6-[1,2,3]triazol-2-yl-phenyl)-methanone) (Bonaventure et al., 2015)
IPSU	OX_2_R selective antagonist	Non-peptide	2-((1H-Indol-3-yl)methyl)-9-(4-methoxypyrimidin-2-yl)-2,9-diazaspiro[5.5]undecan-1-one
MK-1064	OX_2_R selective antagonist	Non-peptide	5-(5-chloropyridin-3-yl)-N-[(5,6-dimethoxypyridin-2-yl)methyl]-2-pyridin-2-ylpyridine-3- carboxamide
MK-3697	OX_2_R selective antagonist	Non-peptide	*N*-[(5,6-dimethoxypyridin-2-yl)methyl]-2-(5-methylpyridin-3-yl)-5-(1,3-thiazol-2-yl)pyridine-4-carboxamide
SDM-878	OX_2_R selective antagonist	Non-peptide	(2-(3-(2-(1H-pyrazol-1-yl)nicotinoyl)-3,8-diazabicyclo[3.2.1]octan-8-yl)-3-methoxyisonicotinonitrile) (Maehara et al., 2020)
LSN2424100	OX_2_R selective antagonist	Non-peptide	*N*-biphenyl-2-yl-4-fluoro-*N*-(1H-imidazol-2-ylmethyl) benzenesulfonamide HCl (Fitch et al., 2014)
MK-4305 (Suvorexant)	Dual receptor antagonist	Non-peptide	[(7R)-4-(5-chloro-1,3-benzoxazol-2-yl)-7-methyl-1,4-diazepan-1-yl][5-methyl-2-(2H-1,2,3-triazol-2-yl)phenyl]methanone
MK-6096 (Filorexant)	Dual receptor antagonist	Non-peptide	[(2R,5R)-5-[(5-fluoropyridin-2-yl)oxymethyl]-2-methylpiperidin-1-yl]-(5-methyl-2-pyrimidin-2- ylphenyl)methanone (Winrow et al., 2012)
E-2006 (Lemborexant)	Dual receptor antagonist	Non-peptide	(1R,2S)-2-[(2,4-dimethylpyrimidin-5-yl)oxymethyl]-2-(3-fluorophenyl)-N-(5-fluoropyridin-2- yl)cyclopropane-1-carboxamide (Yoshida et al., 2015)
SB-649868	Dual receptor antagonist	Non-peptide	N-([(2S)-1-([5-(4-fluorophenyl)-2-methyl-4-thiazolyl]carbonyl)-2-piperidinyl]methyl)-4-benzofurancarboxamide
TCS-1102	Dual receptor antagonist	Non-peptide	N-[1,1′-Biphenyl]-2-yl-1-[2-[(1-methyl-1H-benzimidazol-2-yl)thio]acetyl-2-pyrrolidinedicarboxamide
ACT-078573 (Almorexant)	Dual receptor antagonist	Non-peptide	(2R)-2-[(1S)- 6,7-Dimethoxy-1-{2-[4-(trifluoromethyl)ph-3,4-dihydroisoquinolin-2(1H)-yl]-N-methyl-2-phenylacetamide
ACT-462206	Dual receptor Antagonist	Non-peptide	(2S)-N-(3,5-dimethylphenyl)-1-(4-methoxyphenyl)sulfonylpyrrolidine-2-carboxamide
ACT-541468 (Daridorexant)	Dual receptor Antagonist	Non-peptide	[(2S)-2-(5-Chloro-4-methyl-1H-benzimidazol-2-yl)-2-methylpyrrolidin-1-yl]-[5-methoxy-2-(triazol-2-yl)phenyl]methanone (Treiber et al., 2017)
**Agonists**			
Orexin A (Hypocretin-1)	Dual receptor endogenous agonist	Peptide	H-DL-Pyr-Pro-Leu-Pro-Asp-Cys(1)-Cys(2)-Arg-Gln-Lys-Thr-Cys(1)-Ser-Cys(2)-Arg-Leu-Tyr-Glu-Leu-Leu-His-Gly-Ala-Gly-Asn-His-Ala-Ala-Gly-Ile-Leu-Thr-Leu-NH_2_
Orexin B (Hypocretin-2)	Dual receptor endogenous agonist	Peptide	H-Arg-Ser-Gly-Pro-Pro-Gly-Leu-Gln-Gly-Arg-Leu-Gln-Arg-Leu-Leu-Gln-Ala-Ser-Gly-Asn-His-Ala-Ala-Gly-Ile-Leu-Thr-Met-NH_2_
[Ala^11^,D-Leu^15^] -OX-B	OX_2_R selective agonist	Peptide	H-Arg-Ser-Gly-Pro-Pro-Gly-Leu-Gln-Gly-Arg-Ala-Gln-Arg-Leu-D-Leu-Gln-Ala-Ser-Gly-Asn-His-Ala-Ala-Gly-Ile-Leu-Thr-Met-NH_2_ (Asahi et al., 2003)
Nag 26	OX_2_R selective agonist	Non-peptide	4′-methoxy-N,N-dimethyl-3′-[N-(3-{[2-(3-methylbenzamido)ethyl]amino}phenyl sulfamoyl]-(1,1′-biphenyl)-3-carboxamide (Nagahara et al., 2015)
YNT-185	OX_2_R selective agonist	Non-peptide	2-(dimethylamino)-N-[2-[3-[[5-[3-(dimethylcarbamoyl)phenyl]-2- methoxyphenyl]sulfonylamino]anilino]ethyl]benzamide
TAK-925	OX_2_R selective agonist	Non-peptide	methyl (2R,3S)-3-[(methylsulfonyl)amino]-2-{[(cis-4-phenylcyclohexyl)oxy]methyl}cpiperidine-1-carboxylate (Yukitake et al., 2019)
Yan 7874	Weak partial non-selective agonist	Non-peptide	1-(3,4-dichlorophenyl)-2-[2-imino-3-(4-methylbenzyl)-2,3-dihydro-1H-benzo[d]imidazol-1-yl]ethan-1-ol (Turku et al., 2017)

From observations on the effects of both ligands on second messenger cascades in CHO cells, Holmqvist et al. (2005) demonstrated that OX-A and OX-B do not exhibit the canonical affinity-efficacy relationship at OX_1_R that had been previously described. While the relative potencies of the ligands were as expected for the Gα_*q*_-mediated cascades, for the Gα_*s*_-mediated cascades the relative potencies of the two ligands were observed to be similar. Tang et al. (2008) also observed biased agonism of OX-A and OX-B in their activation of G proteins in HEK293 cells stably expressing OX_2_R. In HEK293 cells, OX-B exhibited bias toward Gα_*q*_-mediated pathways while OX-A seemed to favor Gα_*s*_- and Gα_*i*_-mediated pathways of ERK activation. Neither OX-A nor OX-B showed total bias to a specific pathway for ERK activation, with activity detected for all three G protein cascades that were screened with both OX-A and OX-B. However, it is also of note that this study utilized dominant-negative G proteins, which have been reported to have non-specific effects (Kukkonen, 2004).

Gorojankina et al. (2007) also noted differences in the action of the orexin ligands within an immortalized cell line of olfactory sensory neuron lineage (Murrell and Hunter, 1999) transfected with OX_1_R-EGFP or OX_2_R-EGFP. In this cell line, OX-B produced a synergistic increase in cAMP production with co-treatment of forskolin, however, this was not observed with treatment of OX-A. This was observed with both receptors.

A differential role for OX-A and OX-B in peripheral tissues, in particular adrenal tissue, was postulated early on, when OX-A but not OX-B stimulation resulted in a concentration-dependent increase in basal cAMP production (Mazzocchi et al., 2001). Initially this was thought to be due to the presence of OX_1_R with only low level detection of OX_2_R (Mazzocchi et al., 2001), however further research conflicted with these findings (Randeva et al., 2001). In contrast, OX-B was observed to be more potent in stimulating IP_3_ production in human testicular membrane preparations (Karteris et al., 2004). While it is suggested this indicates a predominantly OX_2_R-mediated effect, no further investigation was conducted.

The possible implications of such differences are highlighted by Patel et al. (2018) in which OX-B, but not OX-A, was found to produce contractile shortening through OX_2_R in cardiomyocytes. It was further observed that OX-B exerted a cardioprotective effect in rat heart models. The G protein-based signaling mechanisms underlying these effects were not thoroughly investigated in this study, however the phosphoinositide 3-kinase/protein kinase B (PI3K/Akt) and ERK1/2/MAPK pathways were implicated as mediators of the OX-B-induced cardioprotective effects observed.

Experiments further investigating these ligand-dependent biases, including effects on other cellular machinery and translation of findings into higher-order cell or tissue environments, would give great insight into the relative roles of OX-A and OX-B.

### Exogenous Ligands

The majority of drug discovery efforts targeting the orexin system have been focused on the development of antagonists as treatments for insomnia and other sleep and mental health disorders. This has led to the development of selective and potent small molecule orexin receptor antagonists as shown in Table 2. Antagonist research has focused on DORAs which have been, at least initially, preferred for clinical applications, since double receptor knockout mice had the most pronounced sleep phenotypes (Mang et al., 2012). However, there is evidence that dual antagonism can promote cataplexy-like symptoms in rodents (Black et al., 2013) and the Food and Drug Administration (FDA) has issued a warning about such events in humans (Farkas, 2013; Hoyer and Jacobson, 2013). Suvorexant and lemborexant are the only orexin-targeted drugs on the market for the treatment of insomnia, with the New Drug Application for Idorsia’s daridorexant (ACT-541468) just being approved by the FDA. Multiple other DORAs and selective orexin receptor antagonists (SORAs), are currently under development/in clinical trials for the treatment of a variety of CNS disorders, such as depression, anxiety, panic or PTSD (Fitch et al., 2014; Bonaventure et al., 2015, 2017; Boss et al., 2020; Kaufmann et al., 2020; Maehara et al., 2020; Zammit et al., 2020b). Alongside the development of antagonists for clinical use, highly selective and potent orexin antagonists have also been produced for research applications as these compounds are highly desirable for probing receptor-specific effects (Nagase et al., 2017).

In contrast to antagonist research that has focused on non-selective compounds, much of the research relating to orexin receptor agonists is focused on OX_2_R-selective agonists. This is in part due to evidence indicating that stimulation of OX_1_R in the ventral tegmental area increases dopaminergic transmission, raising concerns that OX_1_R-targeted agonists may result in dependency issues (Nakamura et al., 2000; Yukitake et al., 2019). For the agonists that have been developed (Nagahara et al., 2015; Turku et al., 2016, 2019; Leino et al., 2018; Rinne et al., 2018), comprehensive analysis of their pharmacological profiles has not been conducted. Studies have mainly relied upon Ca^2+^ measurements to determine receptor selectivity and agonist activity, since until recently, FLIPR has been a traditional workhorse for drug screening in industry. Given the apparent differences between OX-A and OX-B at both orexin receptors, detailed pharmacological profiling of these new synthetic agonists will hopefully assist in developing our understanding of the pharmacological function of the orexin receptors.

Initial reports indicate further optimization may be needed before some of the proposed agonists are suitable for clinical applications (Irukayama-Tomobe et al., 2017), however the OX_2_R-selective agonist YNT-185 has shown positive results in ameliorating morphine-induced sedation without affecting analgesia in a rat model (Toyama et al., 2018). Additionally, the OX_2_R-selective agonist TAK-925 has shown wake-promoting effects in wild-type mice, but not OX_2_R-knockout mice, indicating target-specific effects in an *in vivo* model (Yukitake et al., 2019) as was expected from original studies performed in a variety of orexin receptor knockout mice (Willie et al., 2003; Mang et al., 2012).

As the orexin system has been demonstrated to stimulate multiple downstream signaling pathways, there is exciting potential for therapeutically harnessing any existing ligand-induced bias. An example of this potential is seen in Yukitake et al. (2019) in which the OX_2_R-selective agonist TAK-925 was characterized using an array of methods.

With respect to phospholipase C activity, TAK-925 and the endogenous ligands had equal potency and maximum response. For cAMP response element-binding protein (CREB) and ERK phosphorylation, the potencies for all three ligands were essentially the same but the maximum response to TAK-925 was somewhat lower. With respect to Ca^2+^ elevation and β-arrestin2 recruitment, the endogenous ligands had equal potency while the potency of TAK-925 was 30–50- fold lower; all ligands had the same maximum response. No data were presented on the relative potency of the agonist on non-Gα_*q*_ mediated effects. As more is revealed about the interplay of multiple G proteins in orexin signaling, the potential for biased agonists may become a particularly interesting consideration for the therapeutic targeting of this system.

The development of orexin-targeted therapeutics, especially agonists, is still in the early stages with much more work needed to explore their therapeutic potential. Interest in orexin-targeted therapeutics has remained strong since initial research began in the early 2000’s. Significant efforts have been made in recent years to produce novel small molecule orexin agonists and antagonists which hold exciting potential for future therapeutics. The translation of these compounds to clinical use has been recently demonstrated with Lemborexant being approved in the US and Japan, and the FDA approving daridorexant in January 2022, as well as other compounds, e.g., seltorexant, being in late stage clinical development (Dauvilliers et al., 2020; Hoyer et al., 2020; Kaufmann et al., 2020; Muehlan et al., 2020a,b; Zammit et al., 2020a,b).

## Conclusion

Research into orexin pharmacology, while fragmented, presents a body of interesting and exciting research, which demonstrates many complex signaling behaviors that could hold vital insights into orexin system function and GPCR pharmacology as a whole. The recent development of various biophysical assay panels (Wan et al., 2018; Avet et al., 2020; Olsen et al., 2020) allows for monitoring of the effect of receptor stimulation on a wide range of GPCR effectors, in live cells and in real-time. Applying technologies such as these to study orexin receptor signaling will allow investigation of orexin receptor pharmacology with greater detail and precision. Indeed, the elucidation of orexin function in peripheral environments, and the development of novel, small molecule ligands in recent years demonstrates that there is undoubtedly much still left to be discovered about this system.

## Author Contributions

All authors listed have made a substantial, direct, and intellectual contribution to the work, and approved it for publication.

## Conflict of Interest

KP is Chief Scientific Advisor to Dimerix, of which he has a shareholding. The remaining authors declare that the research was conducted in the absence of any commercial or financial relationships that could be construed as a potential conflict of interest.

## Publisher’s Note

All claims expressed in this article are solely those of the authors and do not necessarily represent those of their affiliated organizations, or those of the publisher, the editors and the reviewers. Any product that may be evaluated in this article, or claim that may be made by its manufacturer, is not guaranteed or endorsed by the publisher.
